# Young Adults’ Perceptions of 2 Publicly Available Digital Resources for Self-injury: Qualitative Study of a Peer Support App and Web-Based Factsheets

**DOI:** 10.2196/41546

**Published:** 2023-01-12

**Authors:** Kaylee Payne Kruzan, Janis Whitlock, Julia Chapman, Aparajita Bhandari, Natalya Bazarova

**Affiliations:** 1 Department of Preventive Medicine Northwestern University Chicago, IL United States; 2 Department of Communication Cornell University Ithaca, NY United States

**Keywords:** nonsuicidal self-injury, self-harm, digital mental health, mobile app, design, intervention, peer support, psychoeducation

## Abstract

**Background:**

Digital resources have the potential to bridge the gaps in mental health services for young people who self-injure. Most research on digital resources for this population has involved observational studies of content in web-based communities or formative studies focused on the design and early evaluation of new interventions. Far less research has sought to understand young people’s experiences with publicly available digital resources or to identify specific components of these resources that are perceived to be of value in their recovery.

**Objective:**

This study aimed to understand young people’s experiences with 2 publicly available digital resources for self-injury—a peer support app and web-based factsheets—and to disentangle potential explanatory mechanisms associated with perceived benefits and harms.

**Methods:**

Participants were 96 individuals (aged 16-25 years) with nonsuicidal self-injury behavior in the past month, who recently completed a pilot randomized controlled trial designed to assess the efficacy of a peer support app as compared with web-based factsheets to reduce self-injury behavior. The trial showed that participants using the peer support app reported less self-injury behavior relative to those receiving the web-based factsheets over 8 weeks. In this study, we used a conventional approach to content analysis of responses to 2 open-ended questions delivered at the end of the trial with the aims of exploring participants’ overall experiences with these resources and identifying the qualities of these resources that were perceived to be beneficial to or harmful for participants’ recovery.

**Results:**

Overall, participants were more likely to report benefits than harms. Participants who used the peer support app reported more harms than those who received the web-based factsheets. In the open coding phase, clear benefits were also derived from repeated weekly surveys about self-injury. Key benefits across digital resources included enhanced self-knowledge, reduction in self-injury activity, increased outreach or informal conversations, improved attitudes toward therapy, improved mood, and feeling supported and less alone. Key challenges included worsened or unchanged self-injury activity, diminished mood, and increased barriers to outreach. The most prominent benefit derived from the web-based factsheets and weekly surveys was improved self-understanding. However, the way this manifested differed, with factsheets providing insight on why participants engage in self-injury and the function it serves them and surveys making the frequency and severity of participants’ behaviors more apparent. The benefits perceived from using the peer support app were general improvements in mood and feeling less alone.

**Conclusions:**

Findings contribute a nuanced understanding of young people’s experiences with these digital resources and have implications for the optimization of existing platforms and the design of novel resources to support individuals who self-injure.

## Introduction

### Background

Nonsuicidal self-injury affects approximately 13% of young adults [1] and as much as 37% of adolescents [2]. Nonsuicidal self-injury refers to self-inflicted damage of body tissue, without suicidal intent. For simplicity, in this paper, we will use the term *self-injury* to refer to nonsuicidal behavior. As a leading risk factor for future suicidal ideation and attempts [3,4], early and effective intervention for self-injury is a public health priority. There are several efficacious treatments for self-injury, but many people do not disclose their self-injury or seek treatment, which limits their access to support and resources that could help [5]. When individuals seek help, it is often through informal supports such as friends or peers in web-based communities [6-8]. Some studies have shown that approximately one-third of young people with a history of self-injury report web-based help seeking, and those engaged in web-based help seeking were younger, endorsed more frequent and recent self-injury behaviors, and reported significantly more suicidal thoughts than those who had not sought help on the web [9]. Thus, digital resources designed for and disseminated in web-based spaces where people engage in self-injury help seeking have the potential to reach a highly distressed and at-risk population [10].

Web-based and mobile digital interventions offer accessible, low-cost, and potentially efficacious ways of bridging the gaps in services for individuals who self-injure and who are not in treatment [11-13]. Although research in this area is nascent, studies support the acceptability and feasibility of digital interventions for self-injury, with some providing preliminary support for their efficacy to reduce the behavior [14]. For example, a pilot trial of an app focused on delivering cognitive behavioral strategies (BlueIce) found that >70% of users reported having stopped or decreased self-harm behavior after 3 months of use [15]. Similarly, Emotion Regulation Individual Therapy for Adolescents—a web-based program focused on the development of emotion regulation strategies—was associated with 69% reduction in self-injury over a 14-week pilot trial [16]. Similar to BlueIce and Emotion Regulation Individual Therapy for Adolescents, most digital interventions incorporate the structured delivery of psychoeducation and strategies or skills grounded in cognitive and behavioral treatment models [14,17,18]. Although the growing evidence in support of self-injury digital intervention is promising, most of the digital interventions developed and rigorously examined are not broadly available to the public. Moreover, little empirical attention has been given to understanding the value of existing publicly available digital resources for self-injury. Resources on the internet are often less focused on the structured delivery of evidence-based strategies and more on psychoeducation, through blogs, websites, peer support, and web-based communities. Whether these existing resources are beneficial for self-injury recovery remains as an open question.

Psychoeducation—an evidence-based intervention focused on increasing a person’s understanding of, and ability to cope with, a condition—is a core component of self-injury prevention and intervention [19-21]. Many websites with self-injury psychoeducation provide basic information on the behavior, possible treatments, and ways of managing the behavior. However, studies focused on how young people perceive and make use of existing web-based psychoeducational materials are sparse, which limits both our ability to draw conclusions about the benefits of such content and to identify any unmet needs that could be addressed through the design and dissemination of digital interventions embedded in spaces where these resources are encountered.

In contrast, web-based communities (eg, forums, social media, and message boards) are a category of digital resources that has received a lot of attention. Studies have shown that people share details about their mental health status and symptoms with peers in web-based communities to obtain support, advice, and validation [22-24]. Studies have also shown that relationships developed between peers in these spaces can decrease feelings of isolation, increase feelings of being understood and accepted, and lead to a great sense of purpose—all factors that are known to contribute to improved mental health and recovery [25,26]. Although many people reap benefit from this exchange of support, observational studies also highlight several risks, including the potential for normalization of the behavior [27,28], narrative reinforcement [29], and exposure to triggering content [8,30,31]. Even with community moderation efforts, people can be exposed to harmful images, texts, or narratives [22,31]. However, these harms are not well understood from the perspectives of individuals actively involved in these communities and are often inferred from static content. Thus, there is a need for studies focused on understanding the specific attributes of web-based communities that are perceived to be beneficial or harmful from the viewpoints of their members.

A study upon which this paper draws aimed to address these gaps in the literature through a small-scale randomized controlled trial of web-based psychoeducation (in the form of factsheets) and an app-based peer support community [32]. Findings showed that participants using the peer support app reported less frequent self-injury and greater confidence to change their behavior over an 8-week period relative to those who received the web-based factsheets. This work provided preliminary support for the value of the peer support app in reducing self-injury behavior. However, what exactly contributed to participants’ experiences with these digital resources and how they perceived these resources to be beneficial or harmful in the context of their recovery remains largely unknown.

### Objectives

This study aimed to understand the relationship between unique intervention components and self-injury behaviors, through the analysis of participants’ experiences with, and perceptions of, these 2 publicly available digital resources for self-injury. We asked the following research questions (RQs):

- RQ1—What are the perceived benefits and harms of engaging with the peer support app and web-based factsheets?
- RQ2—Which features or interactions are perceived to be central to users’ experiences and the benefits and harms derived from use?

## Methods

### Overview

In this study, we analyzed open-ended feedback from adolescents and young adults (aged 16-25 years) regarding their experiences with using the peer support app or receiving web-based factsheets following a 2-month, 2-arm, pilot randomized controlled trial. A detailed description of trial procedures is published elsewhere [32]. In brief, 131 participants were involved in the trial. Half (67/131, 51.1%) of the participants were asked to use the peer support app, TalkLife, which allowed users to make a profile, post, and comment on others’ posts. The other half (64/131, 48.9%) received web-based factsheets, which were publicly available through academic websites on recovery from self-injury, through their email. In addition to engaging with 1 of these 2 digital resources over the course of 2 months, all participants (131/131, 100%) were asked to respond to weekly surveys sent via email to assess their self-injury frequency, urges, and potential mechanisms of change (eg, stigma and sense of belonging). All (131/131, 100%) participants were invited to provide feedback in an open text–based format through the final trial survey. Open-ended prompts were intentionally broad, asking participants about their experiences with using the peer support app or receiving the factsheets, to allow them to comment on any aspect of their experience that was salient. In total, 73.3% (96/131) of the participants responded to at least one open-ended prompt and were included in the present analyses. This included 47% (45/96) of the participants who used the peer support app and 53% (51/96) of the participants who received the web-based factsheets.

### Digital Resources

#### Peer Support App

The first group of participants was invited to use TalkLife (the *peer support app*), which is publicly available for download across app stores. TalkLife is an existing mobile app, not designed by the research team, and is intended to connect young people to exchange support and share stories on topics related to well-being and mental health. The functionality of the peer support app resembles that of other popular web-based communities and social media sites. For example, the app itself has a relatively simple format, allowing users to post and comment on a live feed and encourage peers through 1-click reactions (eg, likes, supports, and hugs). Furthermore, the app allows users to select *categories* for their posts (eg, self-harm, eating disorders, and relationships), which they can use to seek specific advice or filter the content they receive in their main feed. To ensure engagement during the course of the trial, participants were asked to post or comment at least 3 times a week.

#### Web-Based Factsheets

The second group of participants received a series of self-injury *factsheets* once weekly through their email. Similar to the peer support app, the factsheets are publicly available resources hosted on 2 websites devoted to self-injury education, advocacy, and outreach: The Cornell Research Program on Self-Injury and Recovery [33], which is led by JW, and The Self-Injury Outreach and Support [34], which is led by Dr Stephen Lewis. Factsheets were chosen by the research team to represent a variety of content from key domains associated with recovery from self-injury. Each included self-injury psychoeducation through noninteractive infographics and information briefs covering topics about self-injury such as self-injury functions, common myths, how to seek help, and how to cope with urges. Participants could open these weekly factsheets on the web or download them for offline use and sharing. Importantly, these resources were selected by the research team because they were publicly available, were free, and may be encountered naturally by those seeking self-injury information on the web. Links to the factsheets are available in Multimedia Appendix 1.

### Recruitment and Participant Eligibility

Participants were recruited via the web through self-injury information websites, professional networks, social media outlets (such as Facebook or Twitter), listservs, and the university recruitment system. Potential participants completed a web-based survey to determine eligibility based on the following criteria: individuals aged between 16 and 25 years, with current (within 3 months) and chronic (>6 episodes in the past year) self-injury history were eligible to participate. Exclusion criteria included the following: recent history of psychosis (>2 weeks of institutionalization in the past year) or current suicidality (operationalized as suicidal thoughts or plans). All individuals who completed the eligibility survey received contact information for the National Suicide Prevention Lifeline (NSPL) call center and chat line. Participants who were screened as ineligible were provided with a message thanking them for their willingness to get involved, letting them know that they were ineligible based on their responses, and containing a link to resources on the Cornell Research Program for Self-Injury website and NSPL contact information. Participants who reported elevated suicidal ideation or plan received an additional pop-up message encouraging them to reach out to someone for support, with hyperlinks to the NSPL embedded within the message for easy access. Eligible participants received an email from the research team, providing them with key information about their participation. Those who provided consent via the web were randomly assigned to engage with the peer support app or the web-based factsheets for the study duration. During onboarding, participants received a welcome email containing several videos explaining the expectations per week, how to register on the platform (where applicable), and details about how and when they would receive compensation. In total, participants were eligible to receive US $90 in the form of Amazon gift cards for completion of weekly surveys throughout the 8-week trial and were compensated based on the number of weekly surveys submitted. Participants received US $10 for the week-8 survey, which was the data set used in the present analysis. More details on the trial methodology are published elsewhere [32].

### Ethics Approval

This study was approved by Cornell University’s institutional review board (approval number 1807008133), with a waiver of parental consent approved for individuals aged 16 to 17 years. All participants provided informed assent or consent before participating in the study activities. Additional questions were built into the web-based assent procedure to ensure that adolescents understood who they could reach out to if they experienced distress (including contact information for the study’s principal investigator [PI] and national crisis resources), what types of activities they would be asked to engage in, and that they had the right to discontinue the study.

### Data Management

The types of data collected as part of this study were also described in detail in the assent and consent form. This included data from activity on the peer support app and survey responses at baseline, during the trial, and at follow-up (which we used in this study). Participants’ privacy and confidentiality were priorities in our study design. As part of the large study, only the research PI and research coordinator had access to information linking participants to their contact information and unique identifier. All identifying information was kept separate from survey and use data in a password-secured computer that was accessible only to the PI and study coordinator. All data presented in this paper were deidentified before analysis. Participant safety was also a priority. All potential participants were provided with a list of mental health resources (mentioned previously) during the eligibility screening. Enrolled participants were provided with these resources at the beginning and end of the study and after each weekly survey.

### Data Analysis

We used a mixed methods analytical approach in this study. We present quantitative analyses mainly to provide a descriptive overview of our participant sample and a general distribution of our codes. We report on participants’ demographics, mental health history, and previous experiences with mental health–focused apps. Qualitative methods were then used to analyze responses to 2 open-ended prompts aimed at eliciting overall impressions about and experiences with the digital resources after 2 months of engagement. As mentioned previously, although there were 131 participants in the original trial, our sample for the present analysis was limited to participants who responded to at least one of the open-ended questions on the week-8 survey. Complete responses were provided by 73.3% (96/131) of the participants (45/96, 47% of the participants who engaged with the peer support app and 51/96, 53% of the participants who engaged with the factsheets). As the open-ended responses to these questions were meant to solicit general comments about user experience, they were merged at the participant level during open coding.

We used a conventional approach for qualitative content analysis, as described by Hsieh and Shannon [35]. Consistent with this approach, codes were defined during the data analysis process. First, 2 authors independently read excerpts line by line and applied open codes to the transcripts, with the 2 RQs in mind. The authors then met to settle on an initial code structure. This involved discussing the applied codes and removing codes that were not often used. Next, an author and a research assistant familiarized themselves with the data and met to discuss the initial code structure. These 2 coders then applied codes independently to a randomly drawn sample of 20 texts to test for general congruence and allow for further discussion and refinement. Again, infrequent codes were reduced, and several codes were combined. The coders then applied the code structure to the remaining data. All coding discrepancies were discussed by coders in detail until consensus was reached. Finally, coders met to organize the codes in a hierarchical structure and to choose and extract example quotes for the presentation of findings. Quantitative analyses were conducted using SPSS (version 25; IBM Corp) [36], and qualitative analyses were conducted using the Dedoose coding software (Dedoose).

## Results

### Participant Characteristics

#### Demographics

In total, 96 participants aged between 16 and 25 (mean 20.26, SD 2.51) years provided feedback on their experiences with the peer support app or web-based factsheets after 8 weeks of use. Of the 96 participants, most (n=64, 67%) identified as women, whereas 21 (22%) participants identified as men, 8 (8%) identified as nonbinary, and 3 (3%) identified with other gender identities (transmasculine, 2-spirit, and questioning). Of the 96 participants, most were from North America (n=66, 68%), 13 (14%) were from the United Kingdom, and 17 (18%) were from the European Union.

#### Mental Health Characteristics

Participants reported diverse mental health histories. As self-injury commonly co-occurs with other mental health conditions, we assessed for common diagnoses, indicators of lifetime self-injury severity, and traumatic life events through a survey at the beginning of the trial. Of the 96 participants, 70 (73%) reported injuring themselves >50 times in their lifetime, whereas 19 (20%) reported injuring between 21 and 50 times and 7 (7%) reported injuring ≤20 times. Of the 96 participants, 90 (94%) reported having another mental health condition. The 2 most prevalent comorbidities were depression (85/96, 89%) and anxiety (69/96, 72%). Finally, 95% (91/96) of the participants reported at least one other distressing or traumatic life experience (eg, emotional abuse, sexual abuse, physical abuse, death of loved one, or familial divorce).

### Technology Use for Mental Health and Their Perceived Efficacy

Participants were asked whether they had engaged with any other digital resources for mental health including apps, web-based communities, or seeking information through other web-based channels. More than half (61/96, 64%) of our sample reported having used other digital resources. Of the 96 participants, 61 (64%) participants reported using web-based communities and 53 (55%) reported using mobile apps for mental health or well-being, when they entered the trial. Of those reporting use, 48% (46/96) and 22% (21/96) of the participants reported sharing or exchanging information about self-injury, respectively. While interacting through these platforms, approximately one-third of the participants reported some degree of anonymity (eg, not using real name or photo; 38/96, 40% for web-based communities and 30/96, 31% for apps). Commonly reported web-based communities were Reddit (25/96, 26%), Discord (8/96, 8%), Facebook Groups (10/96, 10%), and Tumblr (6/96, 6%). Commonly reported apps were Mood path (7/96, 7%), Calm Harm (11/96, 11%), and 7 Cups of Tea (7/96, 7%). On a scale of 1 to 100, participants rated the overall satisfaction with web-based communities (mean score 21.37, SD 26.24) and apps (mean score 20.49, SD 28.61) as quite low.

### Qualitative Findings

#### Overview

To understand participants’ experiences with these digital resources, we first examined codes and their general distribution across groups. It became evident in our early open coding that the weekly surveys on self-injury activity, which were received by all participants throughout the trial period, were also perceived to be beneficial in fostering reflection. As self-monitoring is a common feature of digital mental health interventions, we chose to include surveys in our coding of benefits and challenges. Findings are organized in two key sections corresponding to our RQs: (1) perceived benefits and challenges associated with the use of digital resources and (2) specific factors or features influencing experience and acceptability.

#### Perceived Benefits and Challenges Associated With the Use of Digital Resources

##### Overview

Participants identified several ways in which the digital resources affected them and their self-injury behavior over the 2-month period. Benefits included references to how the digital resources were perceived to be helpful in their self-injury self-management and included (1) enhanced self-knowledge, (2) reduction in self-injury activity, (3) increased outreach or informal conversations, (4) improved attitudes toward therapy, (5) improved mood, and (6) feeling supported and less alone. Challenges referred to ways in which the digital resources were less helpful or even harmful to participants’ self-injury self-management. These included experiences of (1) worsened or unchanged self-injury activity, (2) diminished mood, and (3) increased barriers to outreach.

To provide a high-level overview of the distribution of codes within our sample, we performed chi-square analyses comparing the benefits and challenges across participants interacting with the peer support app and factsheets. We found that participants who used the peer support app were less likely to mention benefits (odds ratio 0.41, 95% CI 0.17-0.97; *P*=.04) and more likely to report challenges encountered (odds ratio 5.29, 95% CI 1.06-26.43; *P=*.04), when compared with the group that received factsheets. However, overall, participants reported more benefits than challenges. Of the 96 participants, 36 (38%) noted at least one benefit, whereas 10 (10%) noted challenges, across both groups. Of the mentions of benefits, 20% (7/35) were attributed to the peer support app, 40% (14/35) were attributed to the psychoeducational materials, and 39% (13/35) were attributed to the weekly surveys. Of the challenges mentioned, 81% (9/12) were attributed to the peer support app, whereas approximately 9% were attributed each to web-based factsheets (1/12) and weekly surveys (1/12). Of the challenges mentioned, 81% were attributed to the peer support app, whereas approximately 9% were attributed each to web-based factsheets and weekly surveys. A more detailed qualitative report of participants’ reflections on their experiences with each digital resource is provided in the following sections.

##### Peer Support App

The most mentioned benefits identified among participants who used the peer support app were improved mood and feeling less alone. Regarding improved mood, participants described that being able to connect with others through the app helped them to better self-regulate and improve their mood. For example, a participant noted the following:

It was really helpful to be able to talk to people online when I was feeling down.

Participant 28

This participant described that in-the-moment use of the app was helpful in managing difficult moods, whereas other participants noted that using the app helped them to get a better sense of how their emotional state shifted over long periods. A participant described that the app helped them “get a better image of my emotions over the last few weeks” (Participant 11). Both in-the-moment mood boosts and developing a deep awareness of emotional patterns were reflected upon as key benefits across participant experiences.

Another commonly mentioned benefit among participants using the app was feeling less alone in their struggles. A sense of connection was derived from interactions with peers on the app and through reading content from peers with shared experiences or characteristics. For example, a participant described the following:

I’ve met some nice people on [the app] and they make me feel less alone.

Participant 12

Many participants attributed feelings of validation and relief to know that they are not alone to their experience on the app. For example, another participant noted the following:

It really helped me to understand there are people who relate to my struggles.

Participant 13

However, not all participants described that their experiences with peers on the app were beneficial. The most common challenges attributed to the use of the peer support app were its impact on participants’ self-injury behavior and worsened mood. Regarding the impact on self-injury behaviors, a participant described that they felt “like my recovery was less likely after spending any significant portion of time on the app” (Participant 14). This participant, and others, described that being exposed to harmful content and peers’ distress made them feel less inclined toward or hopeful about their ability to recover. Other participants described a more agnostic experience with the app and the impact it had on their self-injury. A participant noted the following:

It didn’t feel like [the app] actively hurt anything, but I’ve relapsed during the course of this survey, so I don’t think it helped either.

Participant 63

In addition, despite some participants describing improved mood owing to engaging with the peer support app, a comparable proportion of users commented on how experiences within the app had harmful effects on their mood. Most of these comments were related to the effects of community negativity. For example, a participant wrote the following:

Most times I felt worse after using the app.

Participant 74

Another participant elaborated upon the following idea:

The toxic community on the app had a very negative effect on my time management and mental health.

Participant 14

Although the fact that there are both benefits and harms perceived from app use is consistent with the existing literature on web-based peer support for self-injury, it is interesting to note how nuanced these effects are, with some participants reporting both benefits and harms after a relatively short period of use.

##### Web-Based Factsheets

The most common perceived benefits of the web-based factsheets were enhanced self-knowledge, reduction in self-injury behaviors, increased outreach, and improved attitudes toward therapy. When participants described improvements in self-knowledge, they commonly referenced factsheets that helped them to explain why they injured themselves or the way self-injury was related to coping. For example, a participant wrote the following:

Learning about why people self-injure helped me gain focus on my coping methods and how compulsive it has become for me when I’m upset.

Participant 16

Similarly, another participant wrote the following:

I liked understanding why self-harm made me feel better and how to change.

Participant 30

This participant also went on to describe how factsheets about the recovery process and readiness to change self-injury behaviors helped to change their perspective on their existing coping strategies:

I feel a lot more normal in coping with urges and understand my thought process in the precontemplation stage in a new light.

Participant 30

Participants also commented on the relationship between the factsheets and their self-injury behavior. Mostly, these comments were regarding the use of new coping skills during the study and how this helped them to reduce their weekly self-injury frequency. For example, a participant wrote the following:

Some of those tips helped me not to injure myself.

Participant 18

Other participants described how simply having new resources made them feel more capable of stopping injury over time and that this was a catalyst for change. Regarding this aspect, a participant wrote the following:

It’s definitely helped me to take the steps towards stopping self harm for good, as I have new coping mechanisms.

Participant 68

Although most comments on the benefits of the factsheets focused on the materials referencing new coping skills, there were also comments on information about help seeking. Several participants noted that the help-seeking materials focused on how to seek help and improved their attitudes and receptivity toward formal treatment. A participant described the following:

I think the pushes for therapy in this study were well placed, because truly, professionals can help those who are troubled better than they can themselves. I’m looking for therapy at the moment.

Participant 19

Another participant similarly noted that materials motivated them to try treatment:

They’ve also helped me be open to begin online therapy, which I will start tomorrow.

Participant 30

In terms of challenges, only 1 participant described how the psychoeducational factsheets did not support their efforts toward recovery from self-injury. In particular, they wrote that a factsheet describing “how to disclose to others” made them feel discouraged about engaging in further outreach, writing that it “made me feel like I was a burden to the only friend I tell about myself injury” (Participant 55). Although most participants described positive experiences with factsheets, this finding underscores the potential harms that come from delivering even well-intentioned materials through a format that lacks context and does not easily facilitate direct paths for follow-up and clarification.

##### Survey

Although we did not set out to explore the survey as a catalyst for benefits and challenges, many participants made explicit reference to the role the weekly survey played in their experience with digital resources. Given the extensive literature on the value of self-monitoring [37-40] and personal tracking in human-computer interaction [41-43], we chose to include comments related to experiences with the survey as they could shed further light on how repeated measurements can be beneficially incorporated into future digital interventions. Similar to the factsheets, the most common benefits attributed to surveys were enhanced self-knowledge, reduced self-injury activity, and improved attitudes toward therapy. Many participants across both groups described that the weekly surveys promoted self-reflection. For example, a participant referred to the survey as a useful check-in:

I liked [the] survey because it gave me an idea of where I was at by the end of every week and allowed me to reflect on my behavior.

Participant 19

Another participant described that the surveys made some of their behavioral patterns more salient:

I liked this survey because it opened my own eyes to some behaviors I hadn’t originally recognized.

Participant 57

In some cases, this enhanced self-awareness motivated participants to make changes to stop or reduce their self-injury behavior. Some participants described a mental shift in how they thought about their self-injury frequency or severity. For example, a participant wrote the following:

The survey helped me pinpoint what could be holding me back from letting go of my addiction to cutting. I felt like I didn’t need to cut as much as normal during this study.

Participant 35

Other participants described that the surveys made them realize that change was needed. For example, a participant wrote the following:

Answering the “how often have you been self harming this week” was very helpful in getting me to realize that I needed to cut back on self harming and/or stop altogether.

Participant 21

Similarly, participants felt empowered to make changes by this newfound awareness. A participant described the following:

The questionnaire is very insightful. I found myself asking questions that get me out of complacency. I gotta start making moves for myself.

Participant 22

Participant 20 wrote that the survey helped them “to reflect on my behaviours and seek active steps and resources to challenge and positively change my behaviours over time.”

Several participants also mentioned improved attitudes toward therapy because of the continuous reflection on the frequency of their self-injury behavior that these surveys provided. For example, a participant wrote the following:

It definitely opened my eyes to see how much I probably need to be in counseling. Having the same questions repeated helped me see how I was growing as well as how I am being held back.

Participant 29

Similar sentiments were evident in another participant’s reflection:

I have never tried therapy, but have contemplated it for a while. These surveys have made me feel, more so than ever, that I should at least try to take the first step.

Participant 40

Although most comments related to the survey were focused on benefits, several participants noted that the survey may have worsened their mood. A participant wrote the following:

I felt somewhat depressed when I answered your questions, but I was okay. It just reminded me of all the things that happened.

Participant 25

Notably, this participant reported that they had seen improvements in their self-injury leading up to the study; therefore, the weekly reflections on self-injury activity may not have been as helpful for them at this point in their recovery.

#### Factors and Features Influencing Experience and Acceptability

##### Overview

In the next section, we delve more deeply into the specific features or interactions that contributed to the benefits and challenges described previously. We present participant quotes in tables and elaborate on particularly novel findings in text. Table 1 includes app features that contributed to perceived benefits, Table 2 includes app features that were associated with challenges, and Table 3 refers to specific content preferences in the factsheets. Participant comments on the factsheets were overwhelmingly positive; therefore, we did not include further reflection on the challenges for this resource. In addition, no corollary comments were made in reference to specific features of the survey. We expand on subthemes with relevance for future design in the following sections.

**Table 1:** Features associated with peer support benefits.

Features	Quotes
Interactions with supportive peers	“I felt fairly supported by responses to any posts or comments I made. I liked the feature where you could react in several different ways alongside commenting, as this was more accessible and the combination helped more people to show support to each other.” [Participant 20]
Sense of shared experience	“Some of the people would comment on my posts and tell me their stories similar to what I would be talking about. Knowing that there are people older than me that understand what I’m going thru really helps and then knowing how their life changed even with the same past also made me feel supported.” [Participant 32]
Space for venting	“Shouting into the void is therapeutic in itself.” [Participant 34]
Immediacy of support	“The fact that there’s almost always a quick response to a post and the responses in general being kind and wholesome added to that feeling of being supported.” [Participant 78]
Anonymity	“It was nice to vent anonymously.” [Participant 36]
Reduced stigma	“Being able to interact with a community of fellow self-harmers without fear of being judged proved itself beneficial to be.” [Participant 40]
Provision of support	“I liked being able to help other people with their problems.” [Participant 39]

**Table 2:** Features associated with peer support challenges.

Features	Quotes
Community negativity	“It made me uncomfortable and felt like an echo chamber of negativity along with people giving instructions on how to cut the ‘right’ way.” [Participant 44]
Interactions with unsupportive peers	“Many of the users on the app made the experience less enjoyable.” [Participant 74]
Perceived inauthenticity of other posters	“Everything feels completely impersonal, and the times I was in crisis and needed help I only really got platitudes in response.” [Participant 63]
Exposure to triggering content	“It can be a very triggering app, yes there are nice people on it that just want to talk but it upset me reading all these upsetting posts.” [Participant 58]
Preference for therapy or in-person support	“I feel like in person connection is more effective than anything online can be.” [Participant 33]
Perceived difficulty in intervening	“It did make me feel less alone but it was not worth having to read through all the other stuff people have going on, I don’t know these people so it makes me sad that they feel like that and there’s not a lot I can do.” [Participant 58]

**Table 3:** References to specific factsheets.

Features	Quotes
Coping strategies	“Learning about why people self injure helped me gain focus on my coping methods and how compulsive it has become for me when I’m upset.” [Participant 16]
Pain offset relief	“Understanding that it is a pain offset not onset that actually helps me feel better.” [Participant 35]
Disclosure	“I liked the guide to self-disclosing about one’s self-injury and I think this is something that will inform future interactions.” [Participant 17]
Science and statistics	“I liked getting science backed info to make it more real.” [Participant 31]
Stages of change	“The last materials in week 8 regarding the different stages and the guide to coping with urges were without doubt the most helpful. I found it helpful for my own identification of struggles with where I’m at now and learned what I could do about it.” [Participant 23]

#### Factors or Features Associated With Peer Support App Benefits

When reflecting on the benefits of using the peer support app, participants generally commented on design features that made the sharing of support possible and qualities inherent in the community of peers. Factors that contributed to benefits included (1) interactions with supportive peers, (2) sense of shared experience, and (3) space for catharsis or venting. Participants also made explicit reference to (1) immediacy of support, (2) anonymity, (3) lack of stigma, and (4) being able to provide support to others to a lesser degree. Table 1 contains quotes corresponding to each of these subthemes.

Consistent with the findings on benefits and challenges related to experiences with the peer support app, participants commonly described characteristics of peers that made them feel accepted and less alone (eg, shared experiences and lack of stigma). Participants similarly appreciated being able to show and receive support in a variety of ways through 1-click responses (eg, likes and hearts) and through commenting on peer posts openly via the main feed and privately via direct messaging. As mentioned previously, participants commonly used the app to obtain feedback or support in moments of distress and thus appreciated being able to receive feedback from peers quickly during these times.

Interestingly, participants described mixed feelings about both venting and provision of support. Although several participants described an intellectual understanding of the benefits of venting, they also expressed dissatisfaction with the experience as a whole. For example, a participant wrote the following:

It is a well-intentioned but likely ineffective app. It’s a good place to vent and send something off into space for the sake of being seen, but often the interactions with others leave much to be desired.

Participant 52

Another participant described the following:

It can be cool I guess if you just wanna vent or talk to people who are going through similar things as you, but there’s way too much negativity there for it to be useful to actually get better.

Participant 42

These participants seemed to feel as if there was a benefit inherent in being able to express themselves openly, but that this may not outweigh exposure to negativity. Similarly, although several participants referred to the benefits of providing support, the balance between support provision and receipt was not always equal. A participant noted the following:

I felt like I supported more people than who supported me.

Participant 58

As is clear in these participants’ experiences, there appears to be a personal *cost-benefit* analysis undertaken to determine the overall value of the resource for their well-being.

#### Factors or Features Associated With Peer Support App Challenges

Participants also described the elements that contributed to challenges or harmful interactions on the app. These elements included (1) community negativity, (2) interactions with unsupportive peers, and (3) perceived poster inauthenticity. Participants also mentioned (1) exposure to triggering content, (2) preference for therapy or in-person support, and (3) perceived difficulty in intervening. Quotes corresponding to these subthemes are presented in Table 2.

In contrast to factors that were helpful, many comments about negative or potentially harmful experiences were related to content, rather than design features, and how participants perceived peers (eg, authentic vs inauthentic). Comments falling under interactions with unsupportive peers or community negativity were differentiated by their reference to isolated incidents resulting in negative interactions with individuals versus a general sense of negativity at the community level. In addition to the quotes in Table 2, there were several references to harassment and trolling. For example, a participant wrote the following:

Whenever I would post I would have people privately message me and try to hit on me. It seems completely inappropriate for this type of app.

Participant 55

Participants also described a perceived inability to effectively intervene while navigating the app, and this led to a sense of helplessness when reading distressing comments from peers. Although many of these comments referred to limitations that participants perceived in their ability to help, there were also comments on the perceived lack of moderation. For example, a participant noted the following:

I understand that everyone should be able to express their feelings, but I feel like if the app itself could somehow catch and delete inappropriate or triggering responses that are meant to be mean to the person, that would be really nice.

Participant 54

Though the app has several layers of moderation, including the ability for users to flag or block harmful peers and administrative moderators that can remove harmful content, this reflects the imperfect nature of top-down and automated moderation efforts on peer support platforms—particularly those devoted to sensitive topics such as self-injury.

#### Preferred Web-Based Factsheets

Participants who received weekly web-based factsheets described specific topic areas that they found to be particularly helpful. Materials related to coping strategies (including distraction techniques) were the most referenced, followed by information on pain offset relief, information on how to disclose, and resources addressing the science behind self-injury and statistics. Quotes corresponding to these content areas are presented in Table 3.

Many participants saw value in factsheets related to coping strategies; however, as in other studies, participants expressed doubt about their ability to use coping strategies when experiencing intense urges [11,13]. For example, a participant wrote the following:

The alternative coping skills have been helpful. However, I still struggle to implement them in moments of very intense frustration, and I still don’t quite know what to do about that.

Participant 17

Factsheets that focused on scientific evidence such as pain offset relief and those including statistics were particularly compelling and appreciated largely because they increased self-understanding and made participants feel as if they were less alone in their struggles.

In addition to the utility of learning about how to disclose, several participants described the factsheets as being catalysts to conversations with others. Overall, 10% (5/51) of the participants described sharing factsheets with family or friends. A participant noted that sharing the factsheets made them feel further supported by their significant other:

My boyfriend has been helping me through a lot of my mental health problems. I discussed some of the articles with him and that made me feel heard and supported.

Participant 30

Another participant described that they opened up to their sister about their self-injury:

I spoke with my sister about what I was learning regarding [self-injury]. I was surprised to find little judgment from her and that alone made me feel better about myself.

Participant 19

In these cases, the materials led to valued offline conversations.

Overall, our findings suggest that there were elements of value in both digital resources, but that they were characterized by different effects and experiences. Several factors were identified as contributing to positive experiences with the peer support app; however, there was also significant variability in the perceived value of features across participant experiences. Individual differences in the types of psychoeducational factsheets that were most valued and useful were also apparent—despite the relative agreement on the importance of learning coping skills. These findings reflect the high level of nuance that individuals perceive when describing their experiences with digital resources and the multifaceted nature of the effects derived.

## Discussion

### Principal Findings

Many of the previous studies on digital resources for self-injury have focused on whether the use of digital interventions leads to more or less of the behavior, with less attention on other types of effects that may be relevant to individuals’ experiences of self-injury and recovery. This study explored the benefits and challenges perceived by young people who engaged with 2 publicly available digital resources for self-injury. Although many of our findings align with the existing literature on the risks and benefits of web-based activities [8,44], most of this literature has inferred these effects from content published within web-based communities and without direct user insight on perceived impacts. Our study makes a novel contribution by directly soliciting impressions from adolescents and young adults after 2 months of engagement with these digital resources. We also begin to disentangle the specific elements and features of these resources that were most valued by users. Collectively, our findings demonstrate that young people perceived benefits from the peer support app, web-based factsheets, and the repeated web-based surveys. All 3 digital resources have the potential for direct and collateral benefits; however, perceptions of their value and limitations differed considerably.

In general, participants were more likely to mention benefits than challenges; however, these varied according to digital resource. Our findings suggest the highest and most consistent satisfaction with the web-based factsheets containing psychoeducation on self-injury and treatment. This finding is not without precedent, as psychoeducation is a core component of self-injury prevention and intervention [19-21,45]. However, in this study, the psychoeducational materials were relatively simple and static, suggesting that even brief factsheets are perceived to be useful. We note that this finding may also reflect the relative simplicity of the factsheets in contrast to the dynamic nature of interactions on the peer support app. For example, there were 58 specific references to features of the peer support app as compared with 29 for the psychoeducational materials (19 for the survey). Engaging in an interactive platform with many affordances and human actors is a very different experience than receiving factsheets directly to your email, to be opened and read at one’s leisure. That is, it may be that the peer support app had potential for more engagement and, thus, greater overall positive and negative impact.

Participants articulated several direct benefits from their experience with the web-based factsheets, with the dominant benefits focused on improved self-understanding, increased motivation to use other coping techniques, and improved attitudes toward additional support. Interestingly, similar benefits were supported through experiences with the survey. However, participants’ reflections on how the survey improved self-knowledge differed from their reflections on the factsheets. Benefits derived from the survey focused on how weekly reflections or *check-ins* made the frequency and severity of participants’ behaviors more apparent. Heightened awareness of their self-injury behavior appeared to increase participants’ intentions to get additional help and engage in coping strategies to reduce self-injury. In contrast, the factsheets appeared to contribute to self-knowledge by providing further explanation for why participants may engage in self-injury and how the behavior serves a particular function. This knowledge itself was helpful for participants and appeared to lead to a newfound understanding of their personal emotional and behavioral patterns. Participants also expressed clear content preferences, with factsheets related to (1) coping, (2) the way self-injury assists in reducing emotional arousal, (3) tips and techniques for productive disclosure, and (4) review of basic science and statistics being the most referenced and perhaps most likely to have an impact on future intervention design.

Both the benefits from the survey and factsheets on self-knowledge resonate with principles of behavioral approaches to self-injury treatment, which emphasize a functional understanding of self-injury and involves tracking antecedents (or triggers) and consequences of the behavior over time. This appreciation of features that allow users to monitor patterns is also consistent with findings from design work on self-injury self-management [11,46] and the large body of literature on the value of self-monitoring in behavior change [40,47,48]. Momentary assessments and passive sensing are increasingly being integrated into mental health interventions [49,50], and our findings suggest the potential of both psychoeducation and regular active or passive monitoring in future digital resources aimed at supporting recovery for individuals who engage in self-injury.

Another possible benefit from the weekly surveys may have been the perceived presence of an empathetic listener. We must acknowledge that the weekly surveys were sent by the study research assistant, and although the emails were meant to be simple reminders, participants may have benefited from regular contact with someone who was responsive and expressed interest in their experience. The potential benefit of having someone bear witness to personal experience was clearly present in comments about the peer support app, and it may have similarly played a role in the surveys. Empathy through repeated contact with someone invested in patient wellness is a critical component of the caring contacts program for suicide prevention. For example, in caring contacts, a clinician sends a series of repeated asynchronous messages to patients following acute care, and this simple intervention has been associated with reduction in suicidal ideation and attempts [51]. Empathetic listening is also a key feature of crisis lines [52]. It may be worthwhile for future digital interventions to consider integrating nonintrusive, low-intensity contact with users, such as through coaching or other automated means [53]. Future studies should explore user comfort with low-intensity contact and the digital formats that are most suitable for this type of intervention.

The main benefits of the peer support app differed from those of the factsheets and weekly surveys and were instead focused on general improvements in mood and feeling less alone. Experiences of interacting with peers seemed to increase the salience of mood and mood shifts for some participants. Other participants mentioned using the app when their mood was particularly low. This finding is consistent with other studies on peer support for self-injury, in that it is common for people to navigate to these apps or communities in moments of distress for just-in-time support [8,27,54,55]. The sense of feeling less alone in one’s struggles through connecting with peers who share their experiences with self-injury was also noted and is aligned with previous studies [56]. However, it is notable that there was little mention of improved self-knowledge or strategies to manage self-injury as a result of using the peer support app. When young people are in a web-based community where they are exposed to others who are in distress without strategies to manage that distress, this may result in negative consequences, including emotional contagion, making them ultimately feel worse [57,58].

Participants described negative effects on mood and interactions or exposure to content that increased or did not help their self-injury behavior. There was significant variability in references to app features, such that some features were identified as being both positive and negative across participants. Examples include anonymity—a feature that was identified as allowing for positive supportive exchanges, while also being a source of negative interaction and perceived inauthenticity of peer exchanges, as in other studies [59,60]. The unique alchemy of the app itself along with individual user characteristics makes assuring that all users have a uniformly positive experience on the platform challenging. Many participants commented on how community negativity outweighed the benefits of sharing experiences with others—describing a cost-benefit analysis inherent in their evaluation of the peer support app. In summary, the challenges that participants experienced with the peer support app underscore the need for additional features and functionality that can mitigate user challenges and amplify user benefits.

### Implications for Developing Future Digital Resources

#### Overview

Most participants reported previous use of digital resources for mental health (eg, web-based communities and apps), suggesting general receptivity to these resources and digital interventions in this population. However, participants also reported low satisfaction with historical use of these resources, indicating a clear need to improve upon existing spaces where individuals seek information and support. Our study contributes additional insights for designers to consider when working to build and improve digital resources for this population. In this section, we outline several implications for design and future studies.

#### Blending Psychoeducation and Peer Support to Amplify Benefits and Reduce Harms

The overall positive experience participants described with the web-based factsheets was notable because many existing websites include psychoeducational content. However, publicly available web-based communities for self-injury do not often include active or structured psychoeducational components. Our study suggests that the integration of psychoeducational components within web-based communities may increase their perceived value and diversify the benefits gained. Web-based communities that include psychoeducation through existing banks or *toolkits* often do so as an additive that is optional to users. For example, 7 Cups of Tea, a digital platform that delivers self-help tools and on-demand emotional support delivered by trained volunteers, has self-help guides with material such as *understanding self-harm behavior* [61]. Other communities, including TalkLife, provide tips for managing self-injury through their blog feature. However, it is not clear how often users access this information, and none of the participants in our study mentioned the presence or use of these offerings as part of their experience with the app. It may be that the current integration of psychoeducation in these web and mobile spaces is not well marketed or prominently displayed or that the materials are not attuned to young users’ needs. Therefore, we suggest a more structured delivery of psychoeducational content.

A way to increase awareness of and engagement with psychoeducation within web-based peer support communities may be to design these materials as a push feature, which encourages or reminds users to perform beneficial actions (eg, read psychoeducation or watch content). We should underscore that there was likely value in receiving materials directly through a prompt (such as email) and with some regularity in this study versus needing to seek out such resources. This may be especially true for individuals with comorbid conditions that affect motivation (eg, depression). In the self-monitoring literature, push elements are frequently more useful and used, relative to pull elements (where users must seek out content) [62,63]. Many of our participants seemed surprised by the benefits they derived from reading the materials, and they may not have had the drive to track this information on their own. Related to this, studies have shown that even among young people who seek information about self-injury on the web, it can be a challenge to access high-quality information grounded in evidence-based practice [64,65]. Therefore, it will be critical for designers to ensure that they are incorporating high-quality psychoeducation within web spaces that are frequently used by young people already, such as web-based communities. Several questions about this integration remain unanswered. For example, before incorporating push elements within an existing web-based community, we need to explore and understand the ethical considerations of such changes and assess community needs. Studies using a user-centered design approach with individuals with lived experience of self-injury and previous experience in using digital resources can help move this line of important research forward.

In addition to making high-quality psychoeducation accessible to individuals engaged in web-based communities, we must also ensure that these materials are relevant and resonate with their experiences [66]. Our study points to several content areas that were particularly valued by participants and helpful in improving self-knowledge and increasing receptivity to additional help, including content on self-injury functions, coping strategies, and available supports (including outreach efforts and therapy). A compelling way to ensure the relevance of psychoeducational content for a diverse set of users may be to use machine learning or decisional logic to tailor the delivery of content to match users’ needs [67,68]. For example, many social platforms send support messages to individuals in crisis based on keyword use, and a similar model may be leveraged to deliver brief psychoeducational interventions. Individuals who post about self-injury may be further prompted with options to receive daily or weekly psychoeducational materials through the platform itself or via email or SMS text message. Individual tailoring could be explored, so that materials are appropriate for users’ existing knowledge, goals, and preferences. For example, early efforts to implement a brief intervention for self-injury through the social media site, Tumblr, have shown that it is feasible to integrate psychoeducation into web-based community spaces [69]. In a study, Tumblr users received a short psychoeducational intervention aimed at decreasing self-injury by targeting self-criticism and hate [69]. Although the results did not show reduction in self-injury behavior, there was evidence for reduction in self-hate and increase in desire to stop self-injury. It may be that cognitive behavioral strategies such as behavioral activation or goal setting may be paired with psychoeducation to increase the likelihood of putting new knowledge into action, thus reducing behavior. In summary, adding value to existing platforms is a worthwhile endeavor because these spaces are already frequently visited by young people who engage in self-injury, many of whom are not otherwise engaged in mental health services.

#### Integrating Peer Presence in Digital Resources for Self-injury

The lack of overlap in benefits perceived across the peer support app, web-based factsheets, and survey suggests the unique ways these resources contribute to recovery. The main benefits of the peer support app appeared to be the sharing of stories, ability to express oneself, and connection to individuals with shared experiences. However, being able to derive benefits was contrasted with harms such as exposure to community negativity that exacerbated mood and self-injury symptoms. This potential for harm may be especially amplified once the novelty of sharing and hearing similar stories wanes. A way to leverage the beneficial qualities of peer support in a different digital context may be to present peer stories alongside psychoeducational programs. Enabling young people to gain exposure to peer experiences in a structured context may circumvent some of the harms inherent in an open (often unmoderated) peer support community, while still providing beneficial qualities to reduce loneliness. Peer narratives are becoming a prominent feature in several brief digital interventions targeting youth mental health [66], including the brief intervention discussed previously [69]. Another way in which these brief interventions leverage peer support is by allowing users who engage in the intervention to share parts of their personal history such as coping strategies, which can have a direct therapeutic benefit, and they can then choose to share this story with future participants. In this way, interventions can crowdsource peer advice and allow young people to learn about others who have a shared experience of self-injury, without being exposed to content that may lead to further distress. Although other psychoeducational websites for suicide and self-injury include peer stories through short video clips, most psychoeducational content that individuals come across on the internet do not include or prominently feature peer stories representing another opportunity to augment existing resources.

In addition to using peer stories as part of digital intervention, researchers and designers should also consider new and safe ways for young people to interact with one another to exchange support. An option may be to build peer pairing or referral systems that connect peers based on a set of characteristics. Creating a structured exchange between 2 individuals may allow users to develop deep connections, while also reducing the likelihood of them being exposed to negative or harmful content. For example, similar to the Alcoholics Anonymous [70] program, peers earlier in their recovery could be paired with peers later in their recovery. Peer pairing would provide users with a dedicated empathetic listener and could be mutually beneficial because studies have shown that being able to help others who have a shared struggle promotes psychological growth [71,72]. Future digital interventions could consider ways of building elements of copresence within interventions—even if it is through a chatbot or low-intensity human support.

#### Promoting Self-knowledge and Encouraging Action Through Design Features

Finally, one of the most consistent benefits that participants derived from the digital resources was an increased sense of self-knowledge. Although this was most apparent in participants’ reflections on their experiences with the web-based factsheets and the survey, it was also highlighted in comments regarding increased awareness of mood patterns while using the app. Given the importance of tracking self-injury behavior using chain and functional analyses in formal treatments for self-injury, it is important to consider ways of improving self-knowledge and increasing awareness through interactions with digital resources. A way to promote awareness is through simple self-report monitoring, such as check-ins that ask users to reflect on a series of questions over a period. Alternatively, passive monitoring can be used to detect patterns and provide a brief report for users along with suggested strategies or activities to improve mental health and reduce in-the-moment distress. Although passive monitoring can relieve the user burden as it does not require participants to input additional data, more active approaches that require users to input data may increase the salience of patterns and allow for more opportunities for self-reflection. To account for different user needs, an ideal design may allow users to switch back and forth between periods of passive and active monitoring (eg, semiautomatic tracking) depending on their needs (as described elsewhere [73]). An intervention incorporating both active and passive monitoring may be especially useful for young people who self-injure because self-injury patterns are not always constant or consistent and long periods can pass between self-injury episodes. A combination of repeated measurements for monitoring can also be leveraged to initiate in-the-moment and tailored interventions.

### Limitations

This study should be considered in the context of several limitations. Although participants were encouraged to respond to open-ended responses honestly, it is important to note that we analyzed self-report data in a study examining the efficacy of a digital intervention. Therefore, desirability bias may play a role in participant responses; however, we feel that the range of positive and negative responses we received is reassuring. In addition, participants’ characteristics may make them willing and interested in using digital resources for self-injury. Our participants may have been more likely to use these resources, given their history of using digital resources and their desire to participate in our trial. This may limit the generalizability of our findings to other populations with less experience with or interest in digital resources and in the context of natural use (outside of a trial). In addition, although we believe that sustained use of the digital resources for 8 weeks is a strength of our study, our results may not extend to more naturalistic use of these resources over long periods. Finally, our results should be interpreted with the understanding that we used a specific set of psychoeducational materials developed by researcher advocates and a peer support platform that was developed as a community for mental health.

### Conclusions

The potential to leverage web spaces to provide resources to support young people who engage in self-injury is an area of increased research interest and practical importance. The unique nature of our study enabled us to focus on the perceived value of 2 publicly available digital resources. In doing so, we discovered details on what is and is not perceived to be helpful for individuals with self-injury history. The general congruence of our findings with themes from other studies exploring the types of services and design features that are valued by individuals with lived experience of self-injury (eg, distraction and coping) provide a clear direction forward for future research and design. Although there is certainly merit in designing new technologies to support individuals who self-injure, there is also a need to improve and augment existing platforms that are already frequently used by these individuals.

## Appendix

Multimedia Appendix 1 Web-based factsheets on self-injury.

## Abbreviations

NSPL: National Suicide Prevention Lifeline
PI: principal investigator
RQ: research question

## Data Availability

The data sets generated and analyzed during this study are available from the corresponding author upon reasonable request.

## References

[1] Swannell SV, Martin GE, Page A, Hasking P, St John NJ (2014). Prevalence of nonsuicidal self-injury in nonclinical samples: systematic review, meta-analysis and meta-regression. Suicide Life Threat Behav.

[2] Jacobson CM, Gould M (2007). The epidemiology and phenomenology of non-suicidal self-injurious behavior among adolescents: a critical review of the literature. Arch Suicide Res.

[3] Kiekens G, Hasking P, Boyes M, Claes L, Mortier P, Auerbach RP, Cuijpers P, Demyttenaere K, Green JG, Kessler RC, Myin-Germeys I, Nock MK, Bruffaerts R (2018). The associations between non-suicidal self-injury and first onset suicidal thoughts and behaviors. J Affect Disord.

[4] Whitlock J, Muehlenkamp J, Eckenrode J, Purington A, Baral Abrams G, Barreira P, Kress V (2013). Nonsuicidal self-injury as a gateway to suicide in young adults. J Adolesc Health.

[5] Muehlenkamp JJ (2006). Empirically supported treatments and general therapy guidelines for non-suicidal self-injury. J Ment Health Couns.

[6] Coulson NS, Bullock E, Rodham K (2017). Exploring the therapeutic affordances of self-harm online support communities: an online survey of members. JMIR Ment Health.

[7] Evans E, Hawton K, Rodham K (2005). In what ways are adolescents who engage in self-harm or experience thoughts of self-harm different in terms of help-seeking, communication and coping strategies?. J Adolesc.

[8] Lewis SP, Seko Y (2016). A double-edged sword: a review of benefits and risks of online nonsuicidal self-injury activities. J Clin Psychol.

[9] Frost M, Casey L (2016). Who seeks help online for self-injury?. Arch Suicide Res.

[10] Hetrick SE, Robinson J, Burge E, Blandon R, Mobilio B, Rice SM, Simmons MB, Alvarez-Jimenez M, Goodrich S, Davey CG (2018). Youth codesign of a mobile phone app to facilitate self-monitoring and management of mood symptoms in young people with major depression, suicidal ideation, and self-harm. JMIR Ment Health.

[11] Honary M, Bell B, Clinch S, Vega J, Kroll L, Sefi A, McNaney R (2020). Shaping the design of smartphone-based interventions for self-harm. Proceedings of the 2020 CHI Conference on Human Factors in Computing Systems.

[12] Stallard P, Porter J, Grist R (2018). A smartphone app (BlueIce) for young people who self-harm: open phase 1 pre-post trial. JMIR Mhealth Uhealth.

[13] Kruzan KP, Whitlock J, Bazarova NN (2021). Examining the relationship between the use of a mobile peer-support app and self-injury outcomes: longitudinal mixed methods study. JMIR Ment Health.

[14] Kruzan KP, Whitlock J, Bazarova NN, Bhandari A, Chapman J (2022). Use of a mobile peer support app among young people with nonsuicidal self-injury: small-scale randomized controlled trial. JMIR Form Res.

[15] (2018). What is self-injury?. International Society for the Study of Self-injury.

[16] Whitlock J, Muehlenkamp J, Purington A, Eckenrode J, Barreira P, Baral Abrams G, Marchell T, Kress V, Girard K, Chin C, Knox K (2011). Nonsuicidal self-injury in a college population: general trends and sex differences. J Am Coll Health.

[17] Washburn JJ, Richardt SL, Styer DM, Gebhardt M, Juzwin KR, Yourek A, Aldridge D (2012). Psychotherapeutic approaches to non-suicidal self-injury in adolescents. Child Adolesc Psychiatry Ment Health.

[18] Arshad U, Gauntlett J, Husain N, Chaudhry N, Taylor PJ, Farhat-Ul-Ain (2020). A systematic review of the evidence supporting mobile- and internet-based psychological interventions for self-harm. Suicide Life Threat Behav.

[19] Witt K, Spittal MJ, Carter G, Pirkis J, Hetrick S, Currier D, Robinson J, Milner A (2017). Effectiveness of online and mobile telephone applications ('apps') for the self-management of suicidal ideation and self-harm: a systematic review and meta-analysis. BMC Psychiatry.

[20] Kruzan KP, Bazarova NN, Whitlock J (2021). Investigating self-injury support solicitations and responses on a mobile peer support application. Proc ACM Hum Comput Interact.

[21] Rodham K, Gavin J, Miles M (2007). I hear, I listen and I care: a qualitative investigation into the function of a self-harm message board. Suicide Life Threat Behav.

[22] Lewis SP, Mahdy JC, Michal NJ, Arbuthnott AE (2014). Googling Self-injury: the state of health information obtained through online searches for self-injury. JAMA Pediatr.

[23] Lewis SP, Rosenrot SA, Messner MA (2012). Seeking validation in unlikely places: the nature of online questions about non-suicidal self-injury. Arch Suicide Res.

[24] Lewis SP, Baker TG (2011). The possible risks of self-injury web sites: a content analysis. Arch Suicide Res.

[25] Swannell S, Martin G, Krysinska K, Kay T, Olsson K, Win A (2014). Cutting on-line: self-injury and the internet. Adv Ment Health.

[26] Whitlock JL, Powers JL, Eckenrode J (2006). The virtual cutting edge: the internet and adolescent self-injury. Dev Psychol.

[27] Andover MS, Schatten HT, Morris BW, Holman CS, Miller IW (2017). An intervention for nonsuicidal self-injury in young adults: a pilot randomized controlled trial. J Consult Clin Psychol.

[28] Glenn CR, Franklin JC, Nock MK (2015). Evidence-based psychosocial treatments for self-injurious thoughts and behaviors in youth. J Clin Child Adolesc Psychol.

[29] Linehan MM (1993). Skills Training Manual for Treating Borderline Personality Disorder: Diagnosis and Treatment of Mental Disorders.

[30] Pritchard TR, Lewis SP, Marcincinova I (2021). Needs of youth posting about nonsuicidal self-injury: a time-trend analysis. J Adolesc Health.

[31] Dyson MP, Hartling L, Shulhan J, Chisholm A, Milne A, Sundar P, Scott SD, Newton AS (2016). A systematic review of social media use to discuss and view deliberate self-harm acts. PLoS One.

[32] Lewis SP, Michal NJ (2016). Start, stop, and continue: preliminary insight into the appeal of self-injury e-communities. J Health Psychol.

[33] Self-injury and Recovery Resources (SIRR). Cornell University College of Human Ecology.

[34] Self-Injury Outreach and Support.

[35] Seko Y, Lewis SP (2016). The self—harmed, visualized, and reblogged: remaking of self-injury narratives on Tumblr. New Media Soc.

[36] Daine K, Hawton K, Singaravelu V, Stewart A, Simkin S, Montgomery P (2013). The power of the web: a systematic review of studies of the influence of the internet on self-harm and suicide in young people. PLoS One.

[37] Marchant A, Hawton K, Stewart A, Montgomery P, Singaravelu V, Lloyd K, Purdy N, Daine K, John A (2017). A systematic review of the relationship between internet use, self-harm and suicidal behaviour in young people: the good, the bad and the unknown. PLoS One.

[38] Smithson J, Sharkey S, Hewis E, Jones R, Emmens T, Ford T, Owens C (2011). Problem presentation and responses on an online forum for young people who self-harm. Discourse Stud.

[39] Whitlock J, Knox KL (2007). The relationship between self-injurious behavior and suicide in a young adult population. Arch Pediatr Adolesc Med.

[40] Arendt F, Scherr S, Romer D (2019). Effects of exposure to self-harm on social media: evidence from a two-wave panel study among young adults. New Media Soc.

[41] Baker TG, Lewis SP (2013). Responses to online photographs of non-suicidal self-injury: a thematic analysis. Arch Suicide Res.

[42] Kruzan KP, Whitlock J, Nesi J, Telzer EH, Prinstein MJ (2022). Digital media, suicide, and self-injury. Handbook of Adolescent Digital Media Use and Mental Health.

[43] Nesi J, Burke TA, Bettis AH, Kudinova AY, Thompson EC, MacPherson HA, Fox KA, Lawrence HR, Thomas SA, Wolff JC, Altemus MK, Soriano S, Liu RT (2021). Social media use and self-injurious thoughts and behaviors: a systematic review and meta-analysis. Clin Psychol Rev.

[44] Birbeck N, Lawson S, Morrissey K, Rapley T, Olivier P (2017). Self harmony: rethinking hackathons to design and critique digital technologies for those affected by self-harm. Proceedings of the 2017 CHI Conference on Human Factors in Computing Systems.

[45] Kruzan KP, Whitlock J, Bazarova NN, Miller KD, Chapman J, Won AS (2020). Supporting self-injury recovery: the potential for virtual reality intervention. Proceedings of the 2020 CHI Conference on Human Factors in Computing Systems.

[46] Grist R, Porter J, Stallard P (2018). Acceptability, use, and safety of a mobile phone app (BlueIce) for young people who self-harm: qualitative study of service users' experience. JMIR Ment Health.

[47] Franklin JC, Fox KR, Franklin CR, Kleiman EM, Ribeiro JD, Jaroszewski AC, Hooley JM, Nock MK (2016). A brief mobile app reduces nonsuicidal and suicidal self-injury: evidence from three randomized controlled trials. J Consult Clin Psychol.

[48] Kruzan KP, Mohr DC, Reddy M (2022). How technologies can support self-injury self-management: perspectives of young adults with lived experience of nonsuicidal self-injury. Front Digit Health.

[49] Braun V, Clarke V (2006). Using thematic analysis in psychology. Qual Res Psychol.

[50] (2020). IBM SPSS Statistics for Macintosh. IBM Corp.

[51] Choe EK, Lee B, Kay M, Pratt W, Kientz JA (2015). SleepTight: low-burden, self-monitoring technology for capturing and reflecting on sleep behaviors. Proceedings of the 2015 ACM International Joint Conference on Pervasive and Ubiquitous Computing.

[52] Khot RA, Aggarwal D, Pennings R, Hjorth L, Mueller FF (2017). EdiPulse: investigating a playful approach to self-monitoring through 3D printed chocolate treats. Proceedings of the 2017 CHI Conference on Human Factors in Computing Systems.

[53] Munson SA, Consolvo S (2012). Exploring goal-setting, rewards, self-monitoring, and sharing to motivate physical activity. Proceedings of the 6th International Conference on Pervasive Computing Technologies for Healthcare (PervasiveHealth) and Workshops.

[54] Murnane EL, Cosley D, Chang P, Guha S, Frank E, Gay G, Matthews M (2016). Self-monitoring practices, attitudes, and needs of individuals with bipolar disorder: implications for the design of technologies to manage mental health. J Am Med Inform Assoc.

[55] Rooksby J, Rost M, Morrison A, Chalmers M (2014). Personal tracking as lived informatics. Proceedings of the SIGCHI Conference on Human Factors in Computing Systems.

[56] Epstein DA, Caraway M, Johnston C, Ping A, Fogarty J, Munson SA (2016). Beyond abandonment to next steps: understanding and designing for life after personal informatics tool use. Proceedings of the 2016 CHI Conference on Human Factors in Computing Systems.

[57] Whooley M, Ploderer B, Gray K (2014). On the integration of self-tracking data amongst quantified self members. Proceedings of the 28th International BCS Human Computer Interaction Conference.

[58] Muehlenkamp JJ, Walsh BW, McDade M (2010). Preventing non-suicidal self-injury in adolescents: the signs of self-injury program. J Youth Adolesc.

[59] Compernolle S, DeSmet A, Poppe L, Crombez G, De Bourdeaudhuij I, Cardon G, van der Ploeg HP, Van Dyck D (2019). Effectiveness of interventions using self-monitoring to reduce sedentary behavior in adults: a systematic review and meta-analysis. Int J Behav Nutr Phys Act.

[60] Rickard N, Arjmand HA, Bakker D, Seabrook E (2016). Development of a mobile phone app to support self-monitoring of emotional well-being: a mental health digital innovation. JMIR Ment Health.

[61] Coppersmith DD, Bentley KH, Kleiman EM, Nock MK (2021). Variability in the functions of nonsuicidal self-injury: evidence from three real-time monitoring studies. Behav Ther.

[62] Cauchard JR, Frey J, Zahrt O, Johnson K, Crum A, Landay JA (2019). The positive impact of push vs pull progress feedback: a 6-week activity tracking study in the wild. Proc ACM Interact Mob Wearable Ubiquitous Technol.

[63] Okeke F, Sobolev M, Estrin D (2018). Towards a framework for mobile behavior change research. Proceedings of the Technology, Mind, and Society.

[64] Kruzan KP, Meyerhoff J, Biernesser C, Goldstein T, Reddy M, Mohr DC (2021). Centering lived experience in developing digital interventions for suicide and self-injurious behaviors: user-centered design approach. JMIR Ment Health.

[65] Dobias ML, Schleider JL, Jans L, Fox KR (2021). An online, single-session intervention for adolescent self-injurious thoughts and behaviors: results from a randomized trial. Behav Res Ther.

[66] Schleider JL, Weisz JR (2017). Little treatments, promising effects? Meta-analysis of single-session interventions for youth psychiatric problems. J Am Acad Child Adolesc Psychiatry.

[67] Smit EM, Linn AJ, van Weert JC (2015). Taking online computer-tailoring forward: the potential of tailoring the message frame and delivery mode of online health behaviour change interventions. Eur Health Psychol.

[68] Bol N, Smit ES, Lustria ML (2020). Tailored health communication: opportunities and challenges in the digital era. Digit Health.

[69] Dobias ML, Morris RR, Schleider JL (2022). Single-session interventions embedded within Tumblr: acceptability, feasibility, and utility study. JMIR Form Res.

[70] Tonigan JS, Connors GJ, Miller WR, Babor TF, Del Boca FK (2003). Participation and involvement in Alcoholics Anonymous. Treatment Matching in Alcoholism.

[71] Riessman F (1965). The “Helper” therapy principle. Soc Work.

[72] Skovholt TM (2016). The client as helper: a means to promote psychological growth. Couns Psychol.

[73] Kruzan KP, Reddy M, Washburn JJ, Mohr DC (2022). Developing a mobile app for young adults with nonsuicidal self-injury: a prototype feedback study. Int J Environ Res Public Health.

