# Supplementary material for: Young Adults’ Perceptions of 2 Publicly Available Digital Resources for Self-injury: Qualitative Study of a Peer Support App and Web-Based Factsheets
Source: JMIR Form Res. 2023 Jan 12;7:e41546. doi: 10.2196/41546 (PMC9880808; doi:10.2196/41546)
Supplement: Multimedia Appendix 1 [file formative_v7i1e41546_app1.pdf]

Psychoeducational factsheets

CRPSIR Resources (selfinjury.bctr.cornell.edu)
Week 1: "Self-Injury Basics Infographic"
Week 2: "How does self-injury change feelings?"
Week 3: "Distraction Techniques and Alternative Coping Strategies Infographic"
Week 4: "Reaching out for help: Talking about ongoing self-injury"
Week 5: "Finding Your Voice: Talking about Self-injury"
Week 6: "Telling family and friends"
Week 7: "Therapy: Myths and misconceptions"
Week 8: "Understanding and using the stages of change model to assess your own progress"

SiOS Resources (http://sioutreach.org/learn-self-injury/if-you-self-injure)
Week 1: "What is Self-Injury"
Week 2: "Am I Alone?"
Week 3: "Why am I Self-Injuring?"
Week 4: "How do I get help?"
Week 5: "What if there is no one to tell?"
Week 6: "What kinds of treatments are there?"
Week 7: "What are some keys to recovery"
Week 8: “A guide to Coping with Urges”

"What is Self-Injury"

Non-suicidal self-injury (NSSI), also referred to as self-injury or self-harm, is the deliberate and direct destruction of one's body tissue, without suicidal intent and not for body modification purposes. Therefore this definition does not include tattooing or piercing, and indirect injury such as substance abuse or eating disorders. Also, this type of self-injury is different than "self-injurious behaviors" (SIB) which are commonly seen among individuals with intellectual and developmental disabilities.

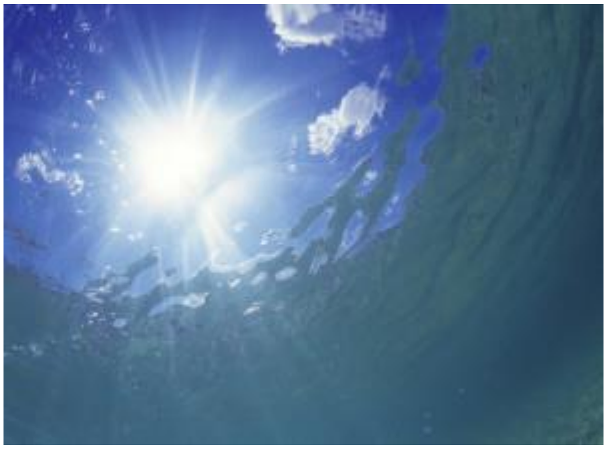

Self-injury Methods

The most common methods of self-injury include cutting, burning, scratching, and bruising. These injuries can range in severity from minor to moderate. Self-injury can start at any age, but most people who self-injure start when they are teenagers. Many people who start self-injuring in their teens continue into adulthood, while others may start self-injuring as adults. Although any one at any age may begin to engage in self-injury, research shows that the most common age of onset for self-injury is early adolescence, more than half of young adults who have engaged in self-injury recall starting at this time, however slightly less than a quarter recall starting before age 12.

Rates

Between 14 to 24% of youth and young adults in the community report engaging in self-injury at least once in their life. Some studies have found even higher percentages if they provide comprehensive checklists of the different types of possible self-injury methods or if they advertise their study as one about self-injury.

14-24% of youth and young adults have self-injured at least once.
One quarter of these have done it many times. You are NOT alone.

Self-injury Outreach & Support (2018 August 31). *What is self-injury?* Retrieved from http://sioutreach.org/learn-self-injury/if-you-self-injure/#ffs-tabbed-11

"Am I Alone?"

Many people who self-injure feel alone and isolated. It is important to know that you are not alone. As noted above self-injury is not uncommon with between 14 and 24 percent of adolescents and young adults reporting that they have self-injured at least once and many of these have done it multiple times.

Why is getting help important?

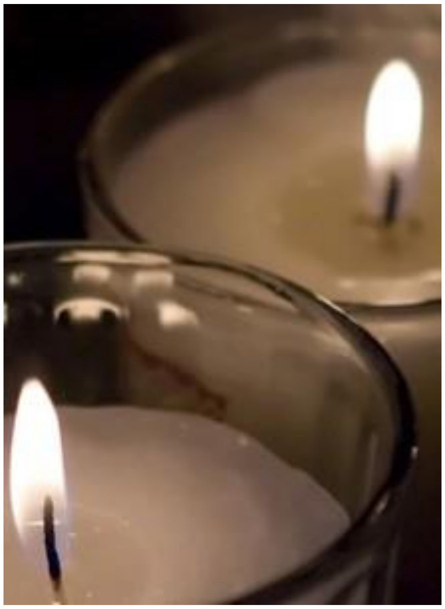

Many people who self-injure report that the more they do it the harder it is to stop. They tell us that it feels like the self-injury has taken over their life.   Self-injury is not a life-sentence, and people can and do stop self-injuring. However, the longer a person self-injures, the more difficult it can be to stop. Some people who used to self-injure have found healthier ways to cope and stopped self-injuring. Some have done this on their own, while others have done it with the support of a professional. In either case it is important to remember that learning new, healthier ways to cope takes time and effort. Don't be discouraged if it doesn't happen right away or if you find it difficult; many people experience this.

This website provides some resources for you in your own efforts and also some materials that you can share with any medical or mental health professional you see, your friend(s), your boyfriend/girlfriend or your family.

All of these people can support you. It is hard to stop — but you **can** stop.

Is there something seriously wrong with me? Am I crazy?

Self-injury is often misunderstood. Some people who hurt themselves may struggle with depression, anxiety, perfectionism, traumatic life experiences or an eating disorder. Others may have none of these difficulties. Self-injuring does not make you crazy. If you self-injure, it does not mean there is something seriously wrong with you but it usually does mean that you are struggling and could use some support.

Many people who self-injure say that the more they do it the harder it is to stop. They tell us that it feels like self-injury has taken over their life.

Self-injury Outreach & Support (2018 August 31). *Am I alone?* Retrieved from http://sioutreach.org/learn-self-injury/if-you-self-injure/#ffs-tabbed-12

"Why am I Self-Injuring?"

The most common reason for self-injury is to **cope with difficult feelings** (e.g., distress, anxiety, stress, sadness). These feelings or thoughts are felt to be so intense and overwhelming that they are intolerable. While it may not be a healthy way to cope, hurting yourself may make you feel better temporarily. This is why the behavior is so difficult to stop.

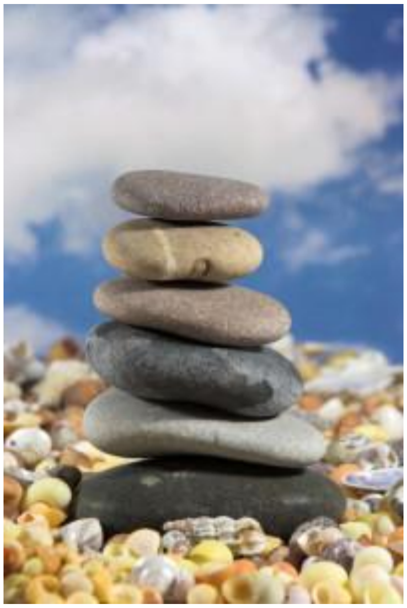

You may self-injure to punish yourself if you **feel you have done something wrong** (even though you may not have actually done anything wrong), or because you feel you have not measured up in some way to your own standards, even though these may be harsh and unrealistic.

You may self-injure to **tell others how you feel**(when it is hard to say it in words). You may self-injure to communicate to someone close to you or the world about your suffering or pain.

Self-injury can also happen when you **feel disconnected from others or even yourself**; it may be used to make you feel something, even if it is pain or to reach out to others.

If you have suicidal thoughts or urges, you may self-injure to **avoid acting on these**.

If none of these reasons sound like they fit for you, there may be other reasons why you self-injure. Everyone has their own reason for engaging in self-injury. Many people say that several of these reasons are true for them, and these reasons can change over time. It is complicated and while others often want to know “Why do you do this?”, it is sometimes difficult to really give one simple reason.

Self-injury Outreach & Support (2018 August 31). *Why am I self-injuring?* Retrieved from http://sioutreach.org/learn-self-injury/if-you-self-injure/#ffs-tabbed-13

"How do I get help?"

How do I know I need help?

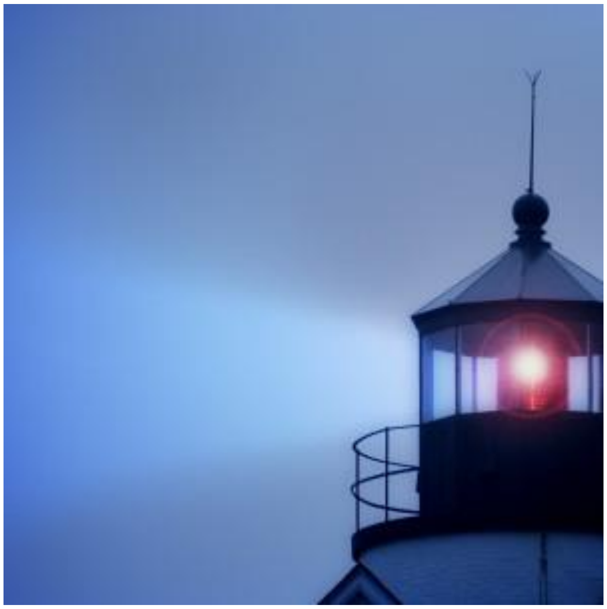

Although many self-injurers say that the self-injury has 'taken over' and they know they need help, others who self-injure feel like they can control the behavior on their own, or stop injuring whenever they want. In either case, hurting yourself is a sign that things are difficult for you. It is a sign that you are not coping well, and that you need to find better ways to cope. Even if you are injuring only sometimes, this suggests that you are having some difficulties with strong emotions and/or communicating with others. Because of this, you might find that getting support is helpful.

How do I tell others who care about me that I'm injuring?

It is common to feel like nobody understands your self-injury. Thinking about telling someone else about your behavior may feel scary, intimidating and/or impossible. Different things may help with some of these worries. Below are some suggestions that may help you feel safer sharing your self-injury with someone else.

1. Identify someone you trust. This may be a parent, aunt, uncle, best friend, teacher, romantic partner or anybody in your life. There is no right answer about WHO can support you best.

2. Write down your story. Sometimes writing how you started self-injuring and what your experience has been like can help you to share it with someone else. This is especially true if you have a hard time talking about your feelings.

Although you want others to react with empathy, understanding, and kindness, this may not always happen right away. Sometimes when people really care about you they can react in ways that may be upsetting and which seem unhelpful. This may be because they don't understand self-injury, find it scary, and do not know what to say or do. They may need some time to understand what you're going through and how to support you best.

3. If you are a teenager and choose to tell a close friend, it is important for your friend to tell an adult. While it may feel like your friend can help you through this, they cannot do it alone. Remember, they are telling an adult because they care about you, not because they aren't a good friend. This can be hard to see in the moment; but it is important for

Self-injury Outreach & Support (2018 August 31). *How do I get help?* Retrieved from http://sioutreach.org/learn-self-injury/if-you-self-injure/#ffs-tabbed-14

you to get the support you need and while a friend can be understanding, they can't do it all alone.

4. Another thing that can be helpful when telling others about your self-injury is to share the General Information Guide from our site. You can either print out or send a link to someone; it can be helpful for that person to learn more about self-injury in order to better understand it.

Self-injury does not mean something is seriously wrong with you
but it often means that you are struggling and could use some support.

Self-injury Outreach & Support (2018 August 31). *How do I get help?* Retrieved from http://sioutreach.org/learn-self-injury/if-you-self-injure/#ffs-tabbed-14

"What if there is no one to tell?"

If you feel like there is nobody in your life who you can tell about self-injury, there are many resources available that can help. You are never alone, and are urged to contact a doctor, a counselor, psychologist or a help line in your area if you feel like there is nobody else you trust to help you. You can refer to the resources at the end of this page for places that may be helpful to you, as well.

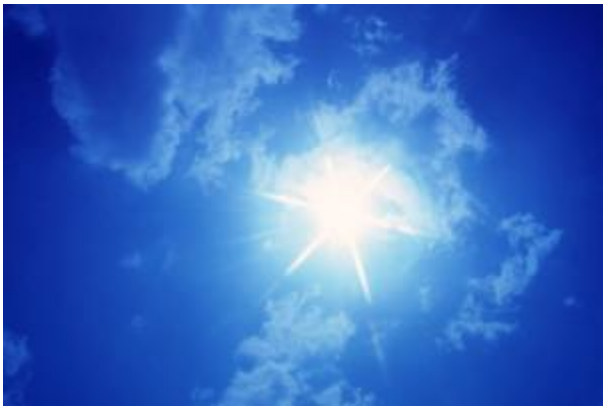

How else can I cope in the moment?

Often, self-injury is used to cope with overwhelming negative feelings and/or thoughts. We have developed a number of coping guidelines which might be helpful:

Click here for a guide about **how to cope with urges.**
Click here for a guide about **how to cope day-to-day.**
Click here for a guide about **how to cope in general.**

Self-injury Outreach & Support (2018 August 31). *What is there is no one to tell?* Retrieved from http://sioutreach.org/learn-self-injury/if-you-self-injure/#ffs-tabbed-15

"What kinds of treatments are there?"

There are some treatments that may be helpful for self-injury. Ideally it is helpful to do this with a professional and support system, but if you don't have that, or feel unable to seek help there are some things you can try on your own to begin coping better. Below, we talk about treatments that you can get from professionals and resources you can use on your own.

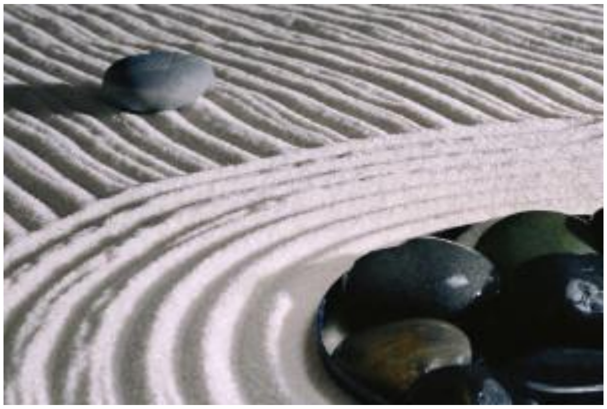

Who treats self-injury?

Licensed mental health professionals are often the people who treat self-injury. This may include: psychologists, psychiatrists, counselors, nurses, and social workers.

Is treatment on my own? Or, in a group?

Treatment can be individual or group-based. Individual treatment will mean that you will work one-on-one with a mental health professional. The professional will take time to get to know you and understand your self injury, and has likely seen many other people with this struggle before.

Treatment can also be group-based. The group sessions will be led by at least one professional, sometimes two.

Will I have to leave my home or be committed to a hospital?

Most often, self-injury can be treated in an outpatient setting. This means you will go to treatment once or twice per week, but will continue on with your regular daily life (e.g., still going to work or school, etc.). If the self-injury is severe or you are at risk of suicide, you may require more intensive treatment, which can happen in an inpatient setting, such as a hospital.

What kind of things happens in treatment?

Treatment for self-injury will often take a few different forms, depending on where you seek help. Two commonly used approaches are:

Cognitive Behavior Therapy (CBT):
CBT is a type of treatment that can help you identify and adjust negative thoughts you may have (e.g., I'm stupid, Nobody loves me) and the feelings that follow from thinking this way (e.g., sadness, pain). Self-injury may be the only way you have to cope now, but CBT focuses on teaching you new ways to cope, including ways to think less negatively. CBT can also help you to cope with urges to self-injury (e.g., what to do instead) and can help you get back involved with things you may have stopped doing because of your self-injury.

Self-injury Outreach & Support (2018 August 31). *What are some keys to recovery?* Retrieved from http://sioutreach.org/learn-self-injury/if-you-self-injure/#ffs-tabbed-16

"What are Some Keys to Recovery?"

In general, research has shown that people who learn healthier ways of coping and who stop self-injuring have done so using one or more of the following key pieces — in either formal treatment or on their own with some other form of support:

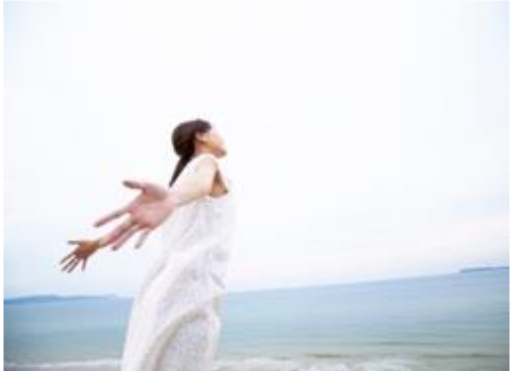

- Learning about self-injury, getting information from reliable sources to better understand your self-injury (see resources below for more information).
- Being really motivated to recover, seeing clearly how the self-injury is negatively affecting your life, seeing the clear. benefits to stopping (for yourself) and believing that it is possible.
- Having at least a few people who know about your efforts to recover, and who are supportive (and know how to support you).
- Working through resources or with a professional to get the skills to manage urges, developing healthy alternative coping skills to replace self-injury, and tolerating/coping with intense emotions.
- Learning how to express emotions and problem solve.
- Developing a new identity as someone who has overcome self-injury.

Self-injury Outreach & Support (2018 August 31). *What are some keys to recovery?* Retrieved from http://sioutreach.org/learn-self-injury/if-you-self-injure/#ffs-tabbed-16
